# The role of policy in supporting environmentally sustainable foodservice in healthcare: lessons from exemplar hospitals

**DOI:** 10.3389/fnut.2023.1122911

**Published:** 2023-07-03

**Authors:** Stefanie Carino, Jorja Collins, Shirin Malekpour, Judi Porter

**Affiliations:** ^1^Department of Nutrition, Dietetics and Food, Monash University, Notting Hill, VIC, Australia; ^2^Department of Dietetics, Eastern Health, Box Hill, VIC, Australia; ^3^Monash Sustainable Development Institute, Monash University, Clayton, VIC, Australia; ^4^School of Exercise and Nutrition Sciences, Institute for Physical Activity and Nutrition, Deakin University, Geelong, VIC, Australia

**Keywords:** foodservice, environmental sustainability, hospitals, policy, healthcare, qualitative

## Abstract

**Background:**

Foodservice in hospitals contributes to the environmental footprint of healthcare delivery. There is little known about the role of policy in supporting environmentally sustainable foodservices. The aim of the study was to explore policy in exemplar environmentally sustainable hospital foodservices from the perspective of hospital staff, toward what makes a policy effective, the limitations of policy, and the influential levels and types of policy.

**Methods:**

A generic qualitative inquiry approach was utilized. Staff involved in foodservices were interviewed about the role of policy during 2020–2021 from 14 hospitals across nine countries. Data were analyzed using framework and thematic analysis.

**Results:**

Policies spanned across high level policies at the level of the healthcare organization, local hospital procedures and protocols, as well as public policy from local, state/provincial and national government. Internal organizational policy was used to embed practices within the organization in the long term and help to build a shared vision and goal where public policy had lacked guidance. The creation, content and methods of communication and creating accountability made internal organizational policy successful. Public policy was most effective when it was mandatory, had clearly defined targets and funding to assist implementation. These exemplar hospitals also demonstrated attributes of policy entrepreneurs by engaging with policy makers to share their stories and lobby government for policy change.

**Discussion:**

Policy from within the healthcare organization is an important mechanism for enabling hospitals to deliver and maintain environmentally sustainable foodservice. Public policy must be designed considering the unique implementation challenges hospitals face to ensure they are successful.

## Introduction

Climate change is an ongoing threat to human health and wellbeing (1). The healthcare sector has a role to play beyond reacting to the impacts of climate change, by adopting proactive measures that build an environmentally sustainable health system from within to ensure ongoing and sustainable health and wellbeing for all. One of the principles critical for achieving an environmentally sustainable health system is to reduce emissions from the delivery of health services (2). Foodservices is an aspect of healthcare delivery that can have vast environmental impacts across the supply chain; from how food is farmed and produced through to how waste is managed (3). The provision of food in hospitals in a safe, nutritious, cost effective, efficient way has, to date, taken priority of over climate change mitigation strategies. There is a need to merge these aspects with a consideration for minimizing environmental impacts as well. The EAT Lancet report outlines the danger of unsustainable food to human and planetary health and calls for a Great Food Transformation, to achieve nutritional requirements within planetary health boundaries (4). There is an array for evidence calling for new policy and system wide change for food system reformation (5).

Action taken by the National Health Service in England to establish a Sustainable Development Unit has provided important learning and insights for driving sustainable healthcare change. One of the influencing variables is the role of governance and regulation. Effective regulatory systems can drive implementation and normalize uptake of sustainable practices more widely, by providing guidance and confidence to those leading change (6). Better understanding and application of governance and regulations reduces their likelihood of limiting innovation in sustainable healthcare. Policy has been described as a formal decision or plan of action adopted by an actor to achieve a particular goal (7). Public policy is the formal decision or plan of action that has been taken by government (7). Policies can facilitate effective integration of climate planning into food production, environment and human health policy (8). For example a law that bans organic waste from entering landfill exists in a number of states and cities in the United States (9). In another example, the Farm to Institution New York State policy aims for government run institutions to spend at least 25% of their food dollars on food grown in New York (10).

Individuals working within hospital foodservice can be effective in driving change (11). However, complex system wide sustainability challenges cannot solely rely on individuals to drive widespread change, rather effective policy and systems are needed. Staff working in hospitals and in foodservices are responsible for adhering to and operationalizing policy. As an example of food policy that has driven practice in this setting, in Australia there is federal legislation requiring food safety programs in foodservice for vulnerable persons in hospitals (12). Foodservice staff are required to design, implement, adhere to and monitor their compliance to meet standards and minimize the incidence of foodborne illness. However, it is common for professionals to feel “policy alienation,” a disconnect from the policies they are responsible for following and implementing (13). It has been established that food is an important part to achieve a zero emissions healthcare sector (14). However, translation of policy ambition into meaningful policy is complex (15). Individuals working in foodservice in hospitals experience firsthand the benefits and challenges of policy to support or inhibit environmentally sustainable foodservice. For this reason, it is important to understand their perspectives toward policies, what is effective and what is not, in order to drive truly effective policy formulation and implementation. Exemplar healthcare facilities who have successfully implemented sustainability initiatives within their organizations provide a unique perspective of the role of policy in driving change. This is an area that has not previously been documented in the literature and is important to be able to support effective policy creation. Therefore, the aim of this study was to explore policy in environmentally sustainable hospital foodservice from the perspective of hospital staff who work in exemplar hospital foodservices. Specifically, the aim was to understand their perspectives about the influential levels and types of policy, the characteristics of effective policy, and the limitations of policy. It is expected that understanding these perspectives will guide advocacy efforts for policy change and considerations to strengthen existing and establish new meaningful policy.

## Methods

This study was approved by the Human Research Ethics Committee (Project ID: 24912).

### Research design

A generic qualitative inquiry approach underpinned by pragmatism was used (16). Research guided by pragmatism seeks a deeper understanding of an issue by drawing on experiences of individuals to guide practical and actionable answers (16). The data used in this study were drawn from a multiple case study exploring institutional drivers of environmentally sustainable hospital foodservices, which concluded there is a need for supportive systems and policies to facilitate widespread change (11).

The focus of the current inquiry was to explore staff experiences and perspectives of policy in driving change. In this analysis, “policy” refers to a formal decision/commitment or plan of action adopted by government or the healthcare organization to achieve sustainable foodservices. This could be “public policy,” those created by government at the national, state/provincial or local level. It could also be “internal organizational policy” such as a strategy, protocol, guideline, procedure or any other relevant document created internally by the hospital. They may relate to any stage of the food supply chain including food procurement, processing, service, disposal of food and related wastes. Contracts between the hospital and external stakeholders, such as group purchasing organizations and central production kitchens were within the scope of “policy” as they typically reference relevant policy and its implementation.

### Participants

Hospitals were recruited purposively. The inclusion criteria for selection required participating hospitals to have clearly demonstrated environmentally sustainable foodservices across any stage of the food supply chain and provided diversity in terms of hospital type and geographical location. They were identified through various mediums such as featured in webinars run by sustainability networks, e.g., Healthcare Without Harm, in literature or reports, listed for receiving relevant awards, such as those by the Soil Association and Practice Greenhealth, and recommended by colleagues active in the field of healthcare foodservice sustainability. The environmentally sustainable foodservice practices were initially identified in these publicly available documents and resources during recruitment, with verification by participants. An online advertisement was also distributed by Healthcare Without Harm and Global Green and Healthy Hospitals to recruit eligible hospitals. Participants were also asked to refer other hospitals they knew of that met the inclusion criteria to allow for snowballing sampling.

Hospital contacts were invited to participate by nominating a staff member with extensive knowledge of the environmentally sustainable foodservice practice/s of the hospital. A senior staff member provided written organizational permission for the staff member to participate in the interview on behalf of the organization. Participants then consented verbally at the beginning of the interview. After the completion of each interview, it was then decided which hospitals to target for further recruitment, in line with the original inclusion criteria. Recruitment continued until the sample of hospitals was deemed as having sufficient “information power.” This was established upon discussion as a research team throughout data collection on whether the study aims could be fulfilled and captured hospitals with a range of practices and from a diversity of contexts and locations (17).

### Data collection

Demographic data were collected verbally and included the following participant information: position title, years in position, employment type and gender. Hospital information was also collected including: size, type of foodservice model and food production and whether it was a public or private facility. Participants were also asked to explain the hospital’s sustainable foodservice practices at the beginning of the interview to verify information previously identified during recruitment (e.g., reports, literature, webinars etc.).

All interviews were conducted between October 2020 and January 2021, using the platform Zoom videoconferencing. The interviewer was an Accredited Practising Dietitian and researcher in environmentally sustainable foodservices. Data were collected via semi structured individual or small group interviews for cases with more than one suitable participant. Interviews were transcribed using Otter.ai., a speech to text transcription application. All transcripts were reviewed for accuracy by the interviewer.

**Figure 1:**
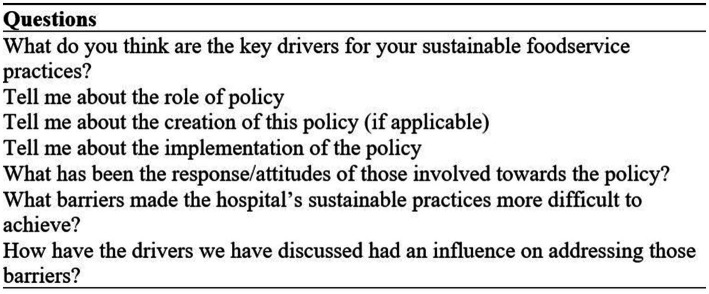
Semi-structured interview questions related to policy [subsection of complete interview protocol previously published (11)].

Interview questions were based around the drivers of the hospitals’ success in achieving sustainable foodservices. A specific series of questions were asked about the role of policy, policy content, creation and implementation. Participants were also asked about challenges they experienced associated with policy and implementation of sustainable foodservice practices. The full interview protocol has been previously published (11) and a summary of the subsection of questions related to policy is presented in Figure 1. Where policy was reported by participants as a challenge, they were asked their perceived reasons. Following the interviews, there was email correspondence with a subgroup of hospitals to obtain policy documents and further information on the policies relevant to their interviews, such as level of governance or to clarify how policies related to each other. Where necessary, policy documents were translated to English language using Google Translate.

### Data analysis

Framework analysis was used initially to create a framework for analysis (18). Firstly, interview transcripts were reviewed to enable the research team to become familiar with participant responses, and a four category framework was developed based on this interview data. Interview transcripts were then coded deductively against these categories as follows: policy levels/types, characteristics of effective policy, limitations of policy, and hospitals acting as policy entrepreneurs. Within these four categories, data were then analyzed using the process of thematic analysis to elicit further meaning from the data (19). This thematic analysis enabled further subthemes to be identified from within the four categories, without a pre-defined framework. Codes were discussed with other researchers and sub-themes were developed.

## Results

### Hospital cases

A total of 14 hospitals were recruited from nine countries (Australia, Singapore, Taiwan, Canada, Denmark, the United States, Austria, the United Kingdom, Netherlands). Further participant demographic details are outlined in Table 1. There were 21 participants interviewed from these hospitals, with more than one participant from some hospitals where two people were identified to be key informants and/or to support adequate communication where participants spoke English as a second language. Their roles included foodservice, dietetics, executive, health promotion and sustainability. Further participant demographic details are outlined in Table 1.

**Table 1:** Participant and hospital demographics (11).

Demographic characteristics	Response	*n* (%)
Participant demographics (*n* = 21)		
Role	Foodservice/hospitality/catering staff	8.5 (40)
	Dietitian	5 (24)
	Hospital executive	2 (10)
	Sustainability staff	2 (10)
	Nutrition director	1 (5)
	Health promotion director	1 (5)
	Community member with specialized knowledge	1 (5)
	Sustainable food coordinator	0.5 (2)
Years in role	0–5	12 (57)
	5–10	3 (14)
	10–15	4 (19)
	15–20	2 (10)
Employment status	Full time	19 (90)
	Part time	2 (10)
Gender	Female	12 (57)
	Male	9 (43)
Hospital demographics (*n* = 14)		
Hospital type	General	12 (86)
	Women and children’s	1 (7)
	General + women’s	1 (7)
Public/private	Public	10 (71)
	Private	3 (21)
	Non-profit	1 (7)
Size (beds)	<50	2 (14)
	51–500	5 (36)
	501–1,000	6 (43)
	1,001–2,500	1 (7)

Examples of sustainable foodservice initiatives implemented within the exemplar facilities were plant based menus, onsite gardens, purchasing of local/seasonal/organic or traditional Indigenous foods, room service model, composting organic food waste, redistributing leftover unserved food to charities and reserving unopened packaged food items.

### Policy levels and types

Responses from interviews revealed the many different policy documents that were influential in sustainable foodservices at these exemplar sites. These consisted of a range of public policies and internal organizational policies. Internal organizational policy included high level guiding documents applicable to the whole healthcare organization such as sustainability commitments, strategic plans and pledges. This then translated into strategies, work plans and specific protocols and instruction documents, for example on how the organization managed food waste, or sustainability criteria for purchasing food.

Public policy documents spanned across national, state/provincial and city levels, standards and strategies. For example, the Denmark Organic Action Plan aimed to increase organic food in public institutions, with the national goal of 60% organic food in public kitchens, set by the Minister for Food, Agriculture, and Fisheries (20). The Ontario Local Food Act was designed to foster resilient local food economies, increase awareness of local food and encourage new markets for local food, by engaging public sector organizations (21).

There were also cases described where policies were associated with grant schemes and programs to support environmentally sustainable foodservice practices. For example, a strategy for public institutions in Quebec to reach a target for local food purchasing had an associated funding program available for projects proposed by institutions to promote and enhance local food in their institution (22).

### Characteristics of effective policy

Participants described several elements of policies which they saw as beneficial in assisting implementation. Firstly, the creation of internal organizational policies and statements were highly valued by participants. They saw it as a way of embedding practices within the organization in the long term and a way to create a shared vision where there was no/little external guidance.

*“We wanted to develop some type of commitment, you know, and kind of create as I call it our legacy, you know, so in five to 10 years perhaps when we're not here, that there was something that we had left behind in terms of, you know, reasons why we did the things we did, and kind of what the outcomes were so it was kind of we measured a lot of the things on a scorecard we did, and like there had to be some positive metrics and things that we were able to prove by doing this kind of project.”* (P7)

Specific characteristics of how these internal policies were created, their content and how they were communicated are outlined in Table 2.

**Table 2:** Characteristics of effective policy as perceived by staff of exemplar hospitals.

Effective policy characteristics	Examples provided by participating exemplar hospitals
**Internal organizational policy**	
*Policy creation* Collaborative creation process involving stakeholders Small scale trial of policy then expanded across the organization Aspiring for systems change	Creating organizational policies was a collaborative process whereby stakeholders were engaged such as foodservice management, manufacturers, distributors, dietitians, patient family advisors There was a desire to work across multiple levels to change the system rather than creating a policy with limited scope.*“The second time around we wanted to make a systems change it wasn’t just about making, you know, adding more local food to a menu which we knew we could do. It was making that system change that was going to impact other health care facilities or group purchasing organization.”* (P7)
*Policy content* Organizational commitment to government requirements and plan for achievement Response to consumer demand Specific targets and frameworks to guide decision making Alignment between levels of internal policy Permission to override existing policy Contracts with suppliers and group purchasing organizations embed sustainability metrics *Accountability and Communication* Effective communication of policy to relevant staff Senior leadership aware of goals, progress and achievements Policy used to communicate requirements and values in decision making and contracts	The focus of some internal documents contextualized requirements of public policy to the organization. *“We have created policies locally here, where we have exceeded the target that we are having from the government concerning the organic. So the government was asking us to reach like a 60% level. So locally, here, we have decided to go for the 95%.”* (P8) Organizations made sure to have specific targets and frameworks to guide action and use in decision making. Effective organizational policy ensured that there was alignment between the levels of documents to ensure vision resulted in action. There were examples of previous policy which restricted sustainable practices and therefore new policy was created to outline the rationale to override existing policy.Documents were made available to staff to ensure accountability. Goals were also shared with senior leadership to hold staff accountable to their responsibilities. *“I think from a sustainability standpoint, the work plans, we have, in particular, really hold people accountable. And we review those with the hospital leadership. And, and people’s progress or lack of progress is documented on those. And so we kind of use that as, as the stick part of that carrot and stick to make sure people will get the things done that they agreed to do, you know, over the period of the year, you know, again, this year is an aberration.”* (P9) Documents were referred to in discussions with contractors and group purchasing organizations as a tool to share the organization’s vision and guide decision making.
**Public policy**	
*Clear targets*	Inclusion of specific targets, such as percentage requirements of local or organic food purchasing, gave hospitals a tangible goal to work toward. An example of this was the 60% organic food in public kitchens goal by the Denmark government (17).
*Funding support*	Associated grants and funding schemes to support hospitals to undertake audits and projects or purchase equipment. For example, in Quebec a strategy had an associated program with funding available for institutions to propose projects to promote local food (19).
*Tax incentives*	These measures incentivized hospitals to make decisions that had better environmental outcomes such as increase in organics and plastics recycling rather than sending to landfill.
*Mandates*	Policy being mandated by government ensured compliance and enhanced the hospitals recognition of the importance of the issue.
*Award schemes*	Associated award schemes for champion organizations encouraged achievements and provided recognition.

In terms of public policy, participants shared several aspects that made them more effective and useful such as having clear targets, and funding support available. These are described in Table 2.

### Limitations of policy

There were aspects of policies reported by participants that limited their ability to implement sustainable foodservices. There was an interplay between internal organizational and public policy whereby hospitals were often bridging gaps in public policy through development of their organizational policy.

However, internal organizational documents were limited in cases where there were no mechanisms for accountability such as key performance indicators. In cases where there were policies and guiding documents, they may not have specifically addressed food. Contracts between the hospital and suppliers or group purchasing organizations often restricted the purchasing of local and Indigenous foods. There was perceived to be a clash in values between those who set up contracts and the users of contracts. For example, valuing price over sustainably produced foods.

Public policy was often targeted at all public institutions in the region, not solely hospitals, and for that reason they were not fit for purpose for the healthcare setting. There were few policies that were mandated but there was a lack of monitoring by government to hold hospitals accountable. For some hospitals, public policy conflicted with existing requirements. For example, a local food act was difficult to follow as trade law required hospitals to not favor local suppliers and purchase from existing contracts only.

*“The policy was there. So we are inspired by it. But in the day to day, it was not easy to follow it because the law was not with us.”* (P6)

Some policies were perceived as too vague to be able to follow and did not have specific targets or guidelines or the support and resources to be able to make changes.

*“There are still no defined targets or anything in terms of you know thou shall procure you know 10% Ontario product for example and there's no reporting it's kind of a voluntary piece.”* (P7)

### Hospitals as policy entrepreneurs

While participants were not specifically asked about their influence on policy, several exemplar sites willingly shared this. These participants could be described as policy entrepreneurs “energetic actors who engage in collaborative efforts to promote policy innovations” (23).

Within the organization, these individuals communicated the link between health and the environment with collaborative working groups of staff. They collaborated with several departments to create a shared vision for sustainable practices to be embedded into the organization’s processes in the long term, such as producing instruction documents and position statements to achieve this.

Externally, these individuals engaged with policy makers to share their success stories. They were part of or led working groups with other hospitals and public institutions to raise awareness of sustainability issues within hospital foodservices and lobby government for policy change.

“*We were trying to educate them in terms of what health care facilities in Ontario had been doing to promote local and so forth in hopes that they would look at our stories and say here's what we've done. And we need you to kind of move this forward so that it does become mandatory for other health care facilities to do that so what we really wanted them to do was a local food act, so it was there, but it wasn't law. And that's what the collaborative group was working on and we had a couple of government officials and the greenbelt fund and so forth that had worked with us on working with the government. We did letters, we shared case studies and all kinds of stuff. And they did end up enacting the local food act which is great*.” (P7)

## Discussion

This research sought to explore policy and the role it played in the experience of international hospitals exemplifying environmentally sustainable foodservices. The findings demonstrate different levels and types of policy, each presenting strengths and limitations in supporting hospitals’ success. The results provide clear insights into how policy can be utilized to serve as an effective driver for sustainable foodservices. The findings suggest that, in the case of these hospitals, public policy does not always support implementation, however, it is effective in prompting hospitals to create their organizational policies. Organizational policies in turn are effective in supporting internal foodservice practice change, through the actions of creating and communicating them and their content. A particularly novel finding of the study was the way in which individuals in these institutions can act as policy entrepreneurs to advocate for policy change.

Unfortunately, inaction can be more significant than action, particularly when older policies have not been updated to reflect current sustainability imperatives and international guidance, or where policies have not been developed to support sustainability initiatives. Despite a previous study about the perspectives of stakeholders working across the hospital food supply chain which suggested that government policy is needed to create change, the present study suggests that the existence of government policy is not always enough to support implementation (24). Instead, policy must be carefully designed to consider implementation.

Policy implementation is described as the actions of individuals or groups directed at the achievement of objectives outlined in policy (25). The policy-implementation gap, whereby policy results in non-achievement, is being increasingly understood to be complex and multifaceted (26). Policy support programs are a new and emerging strategy that has been posed as a way to achieve policy success (26). This requires policy design preparation to understand the problem, policy tracking such as developing targets and progress reports, implementation support such as assistance from experts, and policy implementation review. The present study identified several examples of governments where strategies and funding programs were aligned, contributing to hospitals’ success. These findings suggest that greater support is needed for implementation if public policy is to move beyond solely putting an issue on the agenda. Howlett describes the need for consideration of implementation to be at the heart of policy making, and insights from implementation studies informing the policy process (27). To do this, there is a need for greater understanding of the system resources required for policy implementation. This must come from leaders within the system as well as external expertise and from government, which are specific to sustainable food strategies. For example, tailored programs and resources for local food purchasing that address the challenges hospitals face.

Another important aspect of hospitals’ ability to implement change is having accountability mechanisms in place. These mechanisms were identified in this study as a helpful function of organizational policy to support sustainable foodservices. Given the value of organizational policies, results suggest that all hospitals should have a framework from high level organizational strategic plans to local level work plans. This requirement could be embedded into healthcare accreditation and guidelines which would increase likelihood of compliance. An example of national leadership to achieve sustainable healthcare is the work of the Greener NHS National Programme responsible for all aspects of the NHS’s work on sustainability (28).

Environmentally sustainable hospital foodservices intersects healthcare policy and food policy. Sustainability in the healthcare sector is growing momentum as recently 50 countries committed to developing climate resilient and low-carbon healthcare systems at United Nations Climate Change Conference in Glasgow (29). Simultaneously, food policy that promotes sustainable food systems has recognized the role of large public institutions, which includes the healthcare sector. With government mandating food policy that applies to all public institutions, there is a risk that the unique barriers faced by hospitals and their staff are not adequately considered. Results of this study are particularly relevant for future implementation as they indicate the need for collaboration between policy makers to ensure alignment across healthcare, agriculture and food systems, rather than a siloed approach which is frequently seen. This includes bridging the gap between healthcare networks, government and food policy experts. There is a need to create spaces for cross sectoral dialogue and collaboration to coordinate synergistic actions across sectors and levels of governance (30). Climate related considerations can be incorporated into planning and policy in two ways. Vertical integration is across governance levels and jurisdictions while horizontal integration is of policy goals across different sectors (31). This is a common concept used in sustainable development and ensures that policy makers from different professional backgrounds engage in a common task. Considering integration in both directions ensures policy coherence and prevents conflicting and misaligned policies, as identified in this study.

When considering the advancement of policy, a critical concept is that of policy entrepreneurs. These are people or groups willing to invest their own resources in the hope of a future return (32). Although it was not a specific aim of the study, there were several hospitals in this study that shared their work as policy entrepreneurs. This requires a unique skill set. For example, transforming policy ideas into innovation is a difficult task requiring strategies that are reliant on policy entrepreneurs’ attributes and skills (23). Attributes can be nurtured, and include ambition, social acuity, credibly, sociability and tenacity (23). While skills can be learned including strategic thinking, team building, collecting evidence, making arguments, engaging audiences, negotiating and networking (23). With these skills and attributes, policy entrepreneurs use strategies such as problem framing, using networks, working with advocacy coalitions, leading by example and scaling up change processes. As described in previous research, there are several different values that drive a hospital to pursue sustainable practices (11). Regardless of the values driving the hospital, policy entrepreneurs have social acuity and skills in defining and framing the problem such that policy actors can align with a shared agenda. This was specifically identified in previous research focused on the role of policy entrepreneurship in the creation of urban food strategy development in two European cities (33). Although the food narrative was different between the two cities and was reliant on existing issues, policy entrepreneur networks played the vital role of creating the narrative and building a shared vision among actors (33). This process of reframing issues to align different actors with a joint agenda is important for hospitals to become engaged in, in order to bring about wider change beyond their hospitals.

Given the unique skillset required to be an effective policy entrepreneur, training and tertiary education should be provided informed by the policy entrepreneur literature, based on the important attributes, skills and strategies, contextualized to the healthcare policy setting. The impact of providing policy entrepreneurship training to nurses working in maternal and child healthcare clinics has been investigated (34). This training had a significant effect on policy entrepreneurship behavior and regardless of the level of self-efficacy participants had prior, after training they were able to turn their intentions into behavior. In this way, bureaucrats who do not normally have formal influence in policy design, can use their extensive knowledge of their field toward improving the design of policy. Alongside this, upskilling hospital staff to identify and be prepared for “policy windows” is important. This is described as when the problem, policy and the political streams align and present a window of opportunity for action to be taken (32).

A recent review of research and training related to healthy and environmentally sustainable food procurement and foodservice identified the gap in education and training that exists for nutrition professionals (35). By filling pre-existing training gaps, both with content related to healthy and environmentally sustainable food systems as well as policy entrepreneurship, healthcare workers can mobilize to transform the system they work within. However, healthcare institutions must thoughtfully consider how to support staff to pursue policy entrepreneurship activities. For example, this could be targeted at individuals who are interested, motivated and in positions suited to policy entrepreneur activities. It could be embedded into their position description to provide accountability and individuals would work collaboratively with working groups internally and external sustainability member organizations to increase likelihood of success.

This study is the first to explore policy from the perspectives of those with lived experience working in hospitals that have successful sustainable foodservices. High quality qualitative research demonstrates internal coherence, the alignment between philosophy, methodology and methods such that research questions can be answered to their full potential (36). This study, underpinned by pragmatism, guided our research question to focus on the experiences and actions of staff in relation to policy (16). This approach is a strength of the study as it provided rich and useful information on the effectiveness and drawbacks of policy and viewpoints to strengthen pre-existing and future policy. While this study explored perspectives, another area which would aid policy advocacy is investigating policy decision making processes, implementation, and impact (25). It is important to note that interviews were conducted during late 2020 to early 2021, toward the end of the first year of the COVID-19 pandemic which limited recruitment. It is likely that the perspectives shared by participants may have referred to practice both prior to the pandemic and during the pandemic, while the healthcare system was in a state of major change and stress. Additionally, the sustainable food practices reported by hospitals could not be verified through observation at each hospital due to international travel restrictions at the time. Another point to consider is that while this research explored the role of policy, interviews were conducted with people involved in foodservices and not with staff specifically involved in policy development and implementation. Obtaining this greater understanding of the policy development and translation process from the perspective of policy makers is an important area for future research.

Policy is a necessary and important lever of changing foodservice delivery in hospitals to become more environmentally sustainable. However the design of policy must be tailored to consider implementation challenges unique to healthcare to ensure that policy is fit for purpose rather than existing as a hindrance. Policy makers aiming to improve sustainability of food systems in public institutions will see their efforts fail if they do not properly account for the factors that contribute to implementation success. National strategy is needed to coordinate state/provincial and local level government with an aligned agenda for sustainable healthcare. In doing so, planetary health will be recognized as vital in sustaining human health.

## Data availability statement

The datasets presented in this article are not readily available because the authors did not receive consent for sharing data. Requests to access the datasets should be directed to jorja.collins@monash.edu.

## Ethics statement

The studies involving human participants were reviewed and approved by the Monash University Human Research Ethics Committee (Project ID: 24912). The patients/participants provided their written informed consent to participate in this study.

## Author contributions

SC undertook investigation and formal analysis and wrote the original draft. JP verified coding and interpretation. All authors contributed to review and editing and involved in conceptualization and methodology.

## Funding

SC was supported by the Australian Government Research Training Program (RTP) Scholarship.

## Conflict of interest

The authors declare that the research was conducted in the absence of any commercial or financial relationships that could be construed as a potential conflict of interest.

## Publisher’s note

All claims expressed in this article are solely those of the authors and do not necessarily represent those of their affiliated organizations, or those of the publisher, the editors and the reviewers. Any product that may be evaluated in this article, or claim that may be made by its manufacturer, is not guaranteed or endorsed by the publisher.
